# Supplementary figures and images for: Rapid and predictable genome evolution across three hybrid ant populations
Source: PLoS Biol. 2022 Dec 20;20(12):e3001914. doi: 10.1371/journal.pbio.3001914 (PMC9767332; doi:10.1371/journal.pbio.3001914)

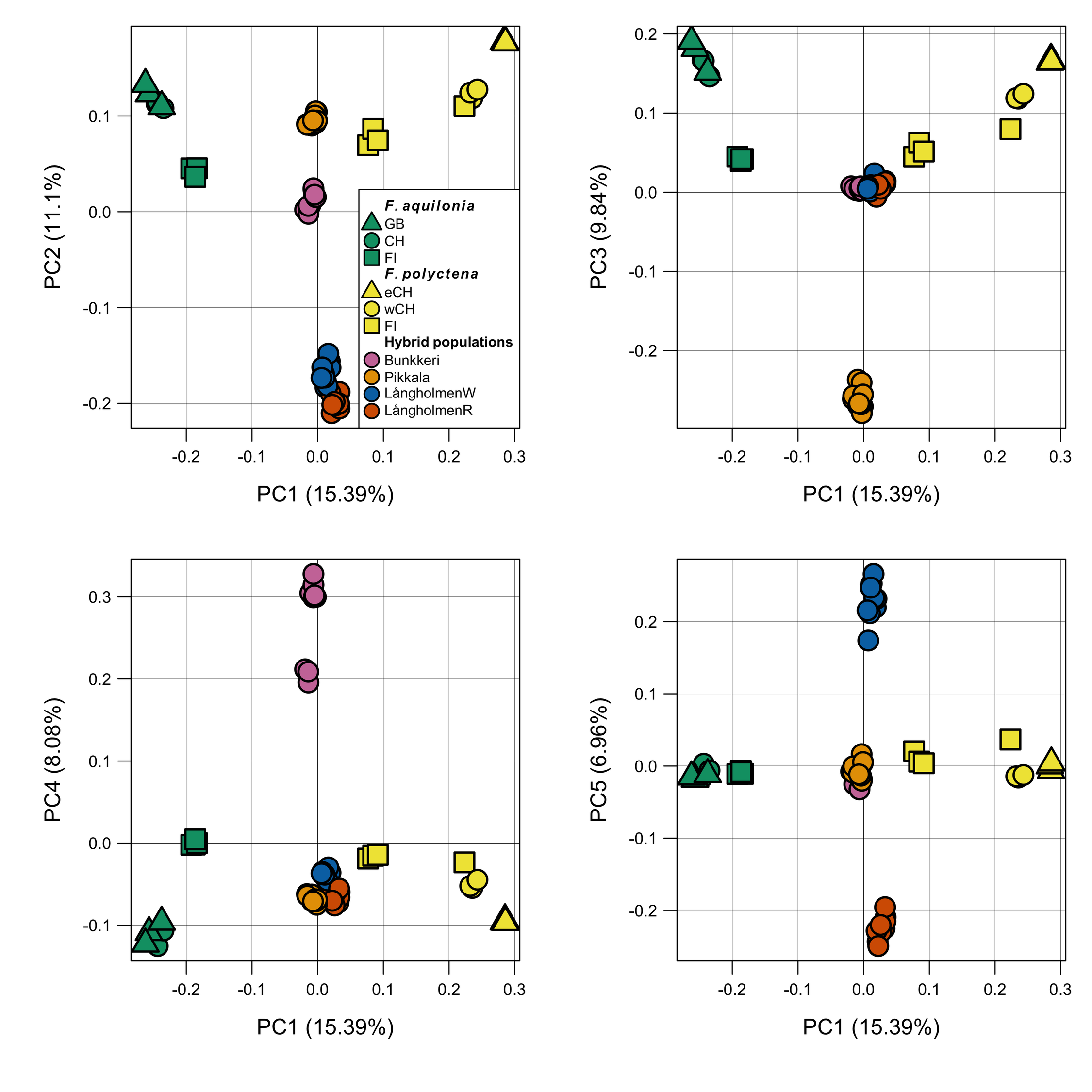

Supplement: S1 Fig — The data underlying this figure can be found in https://doi.org/10.6084/m9.figshare.c.6140793.v3. (TIF) [file pbio.3001914.s001.tif]

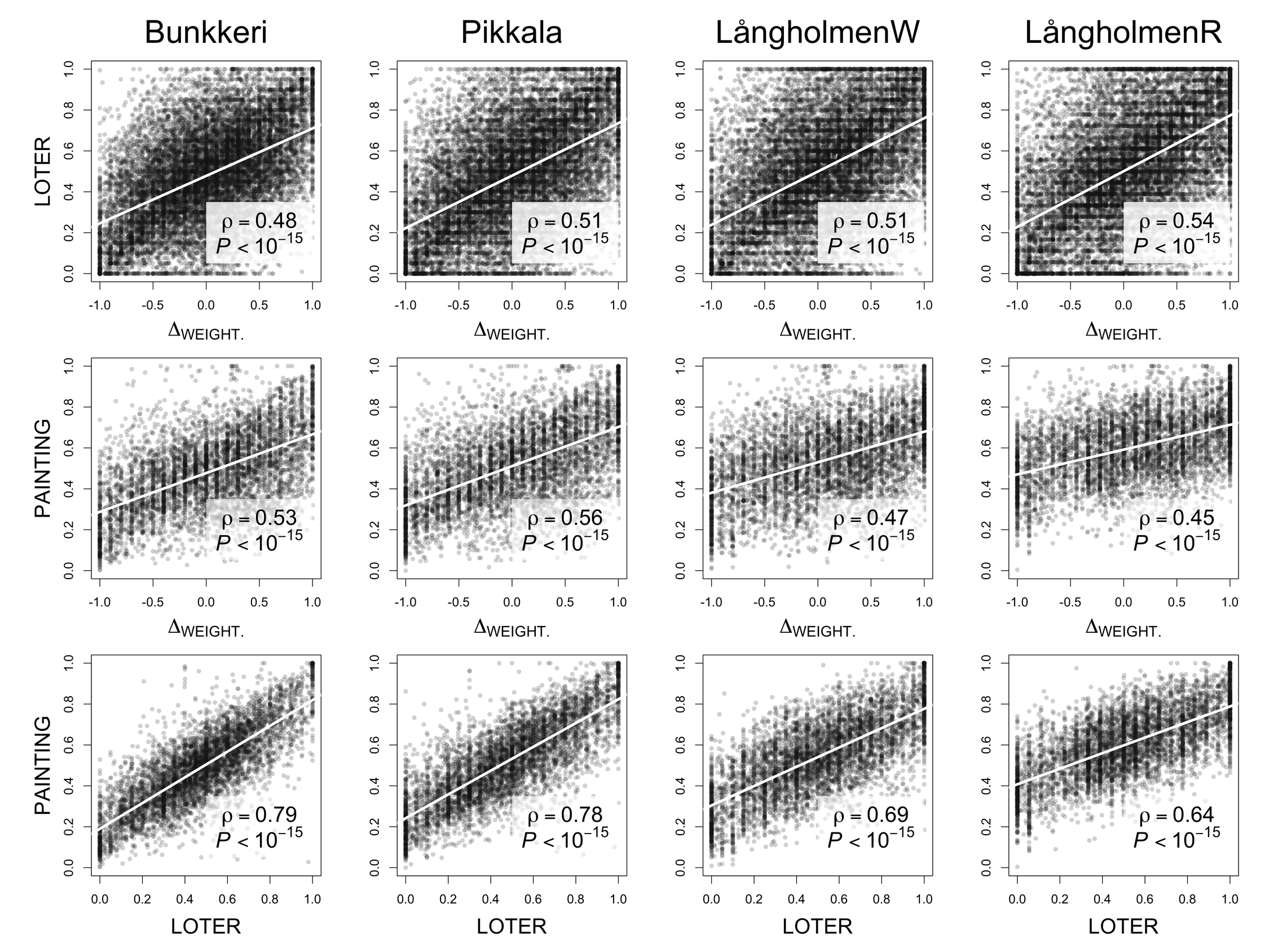

Supplement: S2 Fig — For each hybrid population (columns) are shown TWISST ΔWEIGHT. statistics vs. LOTER local ancestry estimates (first row, 14,890 100-SNP windows), TWISST ΔWEIGHT. statistics vs. naive chromosome painting local ancestry estimates (PAINTING, second row, 5,529 windows with at least 5 ancestry-informative SNPs), and LOTER vs. naive chromosome painting local ancestry estimates (third row, 5,529 windows with at least 5 ancestry-informative SNPs). ΔWEIGHT. ranges between −1 if all topologies in the window group the hybrid population with F. aquilonia, to +1 if with F. polyctena. LOTER and naive chromosome painting are both SNP-based (results averaged over windows) and code ancestries as 0 for F. aquilonia and 1 for F. polyctena. In each panel, the regression line is indicated in white. ρ, Spearman’s correlation coefficient and P, P-value of the Spearman’s correlation test. The data underlying this figure can be found in https://doi.org/10.6084/m9.figshare.c.6140793.v3. (TIF) [file pbio.3001914.s002.tif]

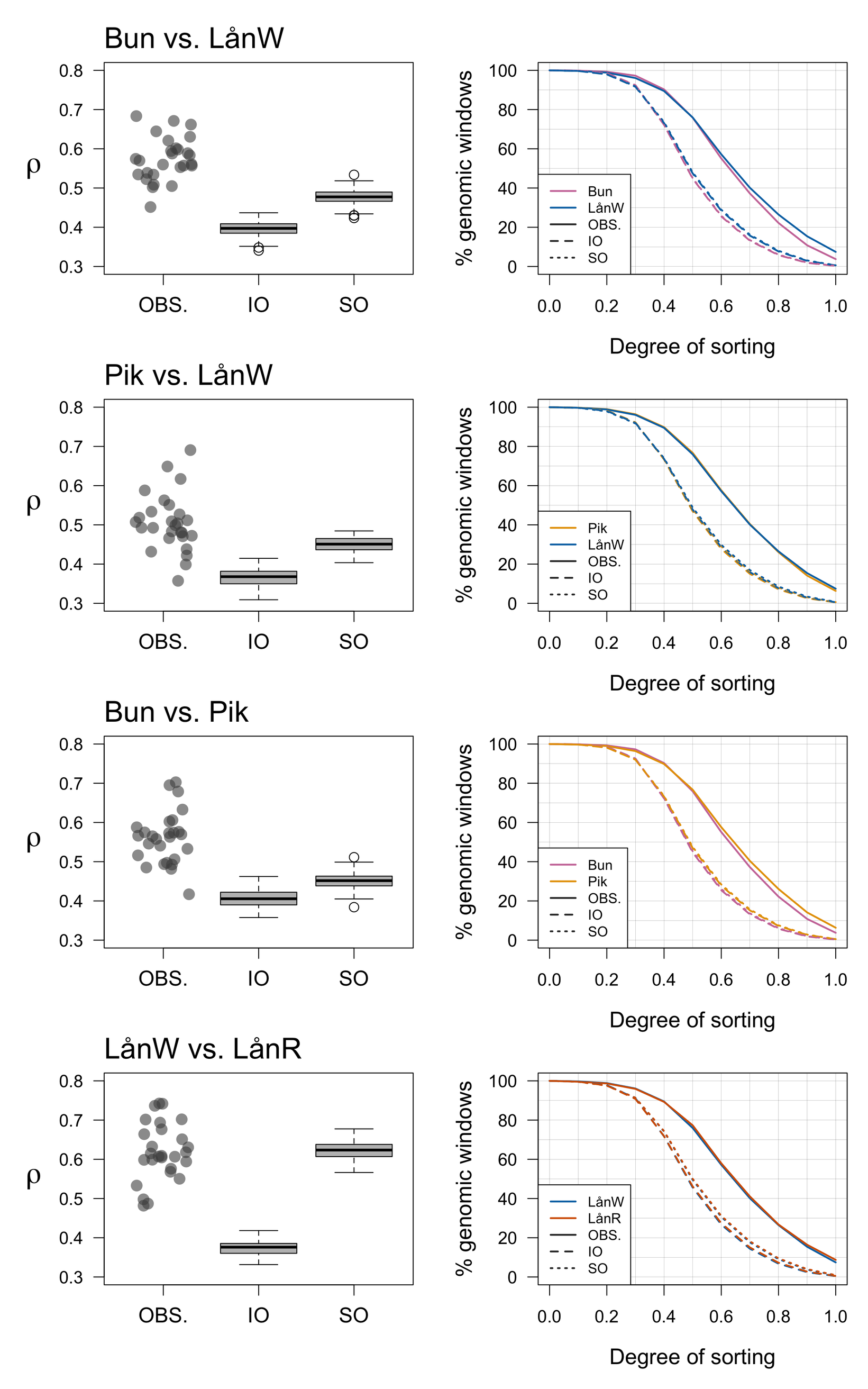

Supplement: S3 Fig — The degree of sorting is measured as the absolute F. aquilonia or F. polyctena weighting. IO: independent origins scenario, SO: single origin scenario (100 independent runs per scenario). The data underlying this figure can be found in https://doi.org/10.6084/m9.figshare.c.6140793.v3. (TIF) [file pbio.3001914.s003.tif]

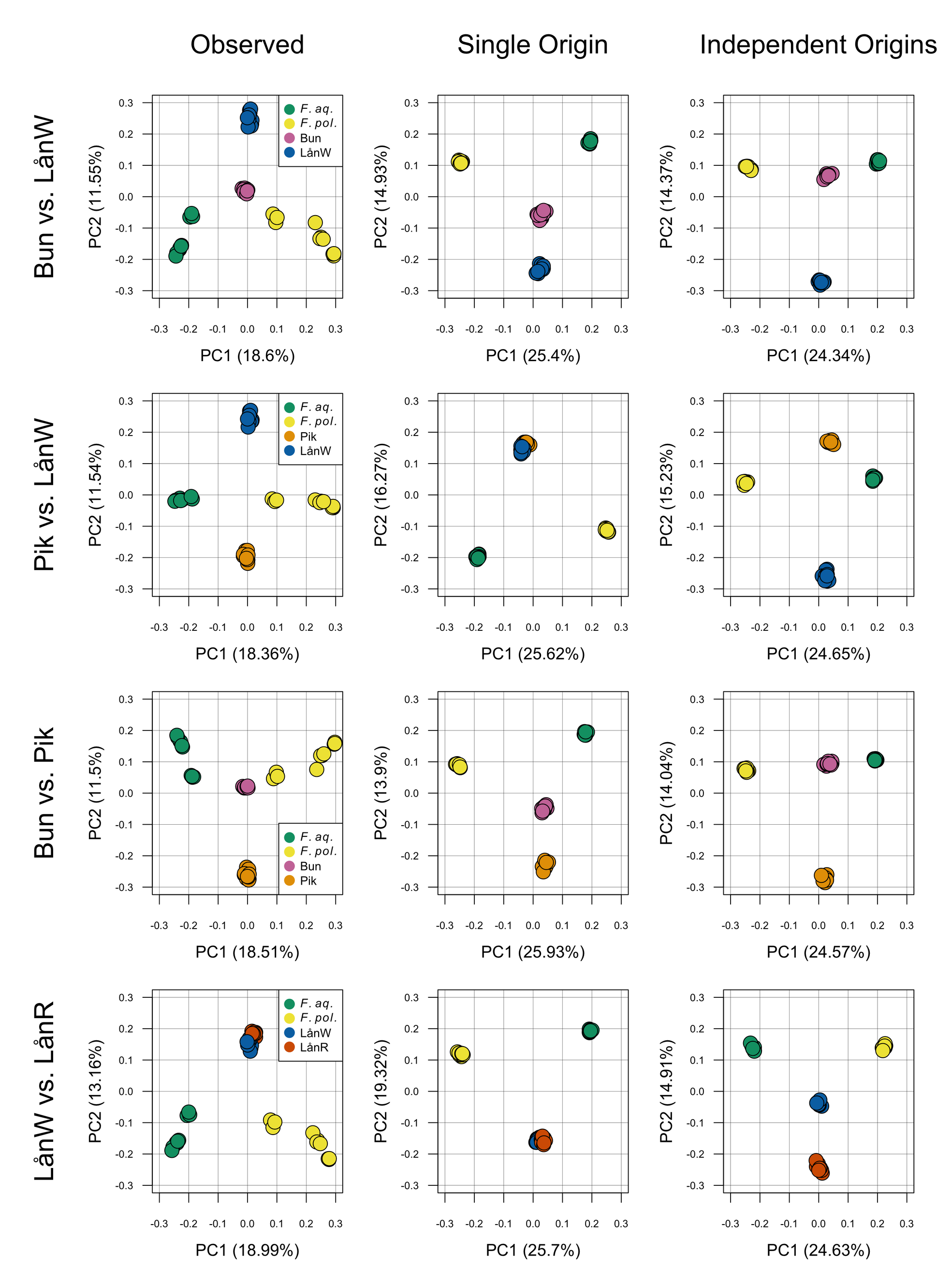

Supplement: S4 Fig — Observed PCAs were obtained as per Fig 1 (5 kb-thinned SNP data, minor allele count ≥2). Simulations were run with msprime using parameter estimates inferred under both single and independent origins scenarios with fastsimcoal2 and assuming a mutation rate of 3.5 × 10^−9^. One run was randomly picked per simulated scenario. The data underlying this figure can be found in https://doi.org/10.6084/m9.figshare.c.6140793.v3. (TIF) [file pbio.3001914.s004.tif]

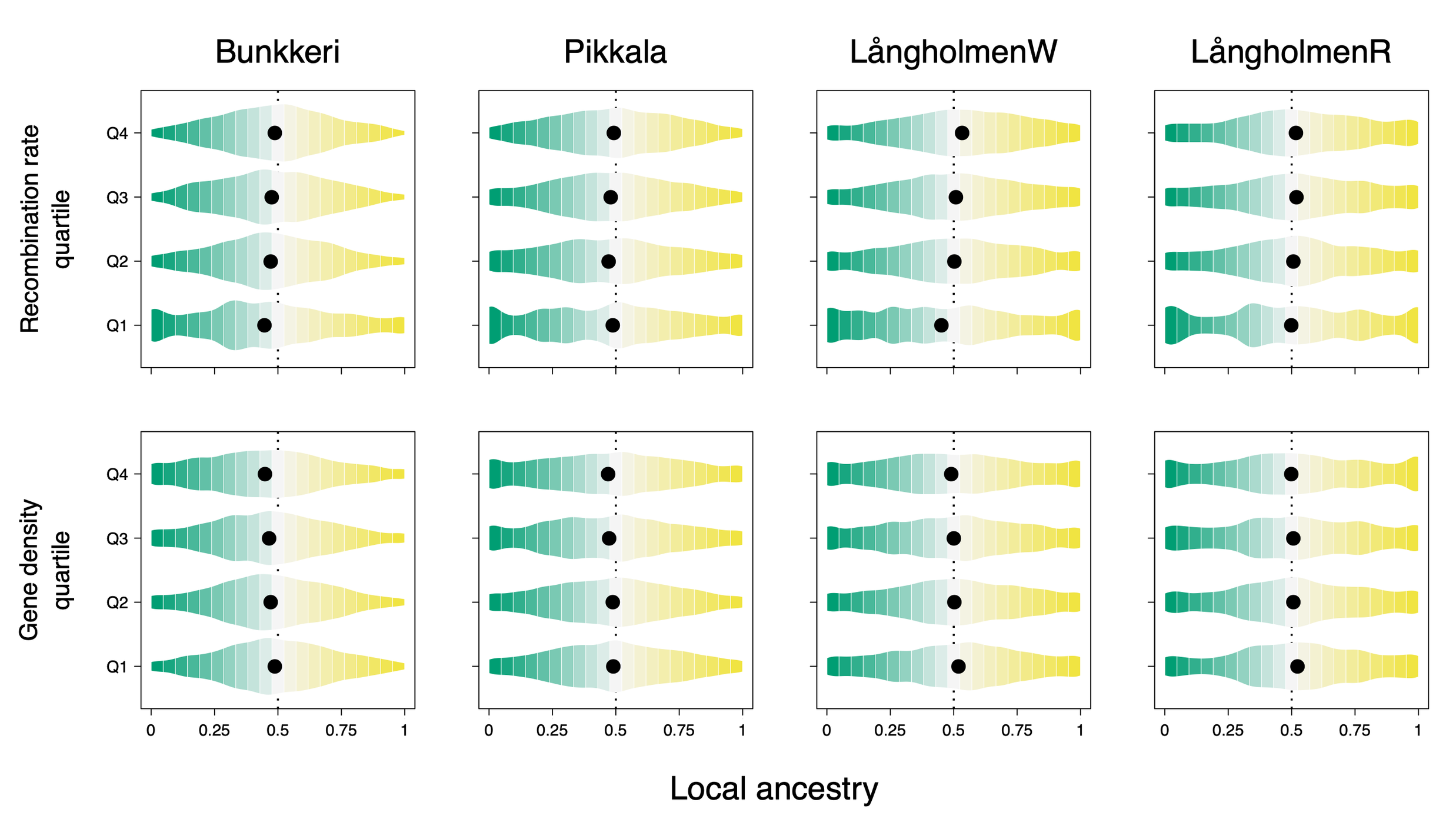

Supplement: S5 Fig — Distribution of LOTER local ancestry estimates (x-axis, 0: fixed for F. aquilonia ancestry component, 1: fixed for F. polyctena ancestry component) across recombination rate (upper row) and gene density (lower row) quartiles in each hybrid population (columns), computed over 20 kbp non-overlapping windows. Medians are indicated with black dots. The data underlying this figure can be found in https://doi.org/10.6084/m9.figshare.c.6140793.v3. (TIF) [file pbio.3001914.s005.tif]
