# Supplementary material for: Rapid and predictable genome evolution across three hybrid ant populations
Source: PLoS Biol. 2022 Dec 20;20(12):e3001914. doi: 10.1371/journal.pbio.3001914 (PMC9767332; doi:10.1371/journal.pbio.3001914)
Supplement: Attachment — Submitted filename: Nouhaud_2022_response.pdf [file pbio.3001914.s019.pdf]

Dear Dr Nouhaud,

Thank you for your patience while your manuscript "Rapid and repeatable genome evolution across three hybrid ant populations" was peer-reviewed at PLOS Biology. It has now been evaluated by the PLOS Biology editors, an Academic Editor with relevant expertise, and by four independent reviewers.

You'll see that while the reviewers are broadly positive about the study, they each raise a number of concerns; strikingly, for example, several of the reviewers think that your evidence for the hybrid populations being truly independent is currently inadequate, and this key issue must be addressed before further consideration.

In light of the reviews, which you will find at the end of this email, we would like to invite you to revise the work to thoroughly address the reviewers' reports.

Given the extent of revision needed, we cannot make a decision about publication until we have seen the revised manuscript and your response to the reviewers' comments. Your revised manuscript is likely to be sent for further evaluation by all or a subset of the reviewers.

We expect to receive your revised manuscript within 3 months. Please email us (plosbiology@plos.org) if you have any questions or concerns, or would like to request an extension.

At this stage, your manuscript remains formally under active consideration at our journal; please notify us by email if you do not intend to submit a revision so that we may withdraw it.

Thank you again for your submission to our journal. We hope that our editorial process has been constructive thus far, and we welcome your feedback at any time. Please don't hesitate to contact us if you have any questions or comments.

Sincerely,

Roli

Roland Roberts
Senior Editor
PLOS Biology
rroberts@plos.org

We thank the Senior Editor and the Reviewers for their overall enthusiasm about our study and for the high quality of their comments. In this revised version we have addressed all the comments made by the Reviewers by implementing new analyses and simulations, and significantly rewriting the manuscript. Main changes to the manuscript are:

- **We provide new evidence for the independent origin of hybrid populations and the contribution of repeated selection in the parallel patterns reported** (new Fig.

1F, results and discussion Lines 107-132, methods L424-438, line numbers refer to the track-changes version). We have implemented an AIC-based model choice procedure to disentangle between single origin (SO) and independent origins (IO) admixture scenarios (key issue raised by the Editor, Reviewer 2 - comment #1, Reviewer 3 - comment #1). This new analysis is conclusive for three out of the four hybrid population pairs analyzed, providing more support towards an IO scenario in two pairs (Bunkkeri - LångholmenW and Bunkkeri - Pikkala) and SO for the third pair (the two sympatric Långholmen lineages). These new results provide stronger evidence towards independent admixture events, and also consistently rule out gene flow between hybrids post-admixture.

- **We show that under neutrality, shared hybrid ancestry cannot explain observed sorting levels** (Fig. 2C-E, S3 Fig.). To provide a more comprehensive view of hybrid genome evolution in the wood ant system under competing hypotheses, we have expanded neutral simulations and now present results of both IO and SO scenarios fitted for each hybrid population pair (Reviewer 4 - comment #2, Reviewer 2 - minor comment). Neutral simulations indicate that correlation levels expected under neutrality are comparable to the ones we observed, but the extent of sorting remains higher with our empirical data than in either the IO or SO neutral simulations (Reviewer 2 - comment #1, S3 Fig.). This suggests that selection drives the sorting of ancestral variation after admixture.
- **We show that our neutral simulations adequately capture the observed polymorphism patterns** in terms of diversity (S12 Table), divergence (S13 Table) and genetic structure (S6 Fig., Reviewer 2 - comment #2). While compiling these new results to answer the Reviewer's comment, we identified an error in our msprime simulations (initially displayed in our previous Fig. 2E). N_e estimates were not rescaled properly (to account for ploidy level differences between fastsimcoal2 and msprime) and initial msprime simulations were run with larger N_e estimates than inferred by fastsimcoal2. This error has been fixed and the conclusions of our study remain unaltered (new Fig. 2E).
- **We provide more biological context about colony establishment in wood ants** in the introduction (L70-82) and we rewrote the results section on mtDNA data (L92-100, Reviewer 1 - comment #3, Reviewer 3 - comment #1). We now discuss mitochondrial patterns in the light of both SO and IO scenarios, and we "suggest" IO as a more likely scenario (L132) given biology of wood ants and model choice results (L126-132).
- **We choose to emphasize the predictability of sorting toward either ancestry component, because this finding holds true regardless of whether SO or IO is true**. However, we still note that IO appears the more likely scenario, which would imply that the sorting is not only predictable but also repeated. We have edited the title of the revised manuscript accordingly, from "rapid and repeatable genome evolution across three hybrid ant populations" to "rapid and predictable genome evolution across three hybrid ant populations".
- We have tested additional demographic models suggested by the Reviewers (Reviewer 3 - comment #2 on excluding sorted regions; Reviewer 4 - comment #1 on the joint analysis of all populations). Results were unfortunately inconclusive because of too little data left after excluding sorted regions, but our new model choice analysis should alleviate the Reviewer's concerns. Likewise, the joint modeling of all populations (minus the LånR lineage) led to convergence issues because of a high

number of parameters to estimate, coupled with the high dimensionality of the joint 5D-SFS with many zero entries. We provide more context and we justify our analysis choices under the respective Reviewer comments.

- Altogether we have added 22 references in the revised version (including all the works suggested by the Reviewers), providing more context in the introduction (L38-47,52,55-61; Reviewer 1 - comment #1, Reviewer #2 - minor comment), and the discussion (L201-239, Reviewer 1 - comment #4). The full, updated reference list can be found in the final version (not the track-changes version, because of PaperPile issues).

In accordance with PLOS' data policy, all data used to draw the conclusions arrived at in our manuscript is freely accessible in appropriate repositories, as indicated in the relevant section:

- FASTQ files are available on ENA under projects PRJEB55288 (hybrid samples) and PRJEB51899 (*F. aquilonia* & *F. polyctena* samples).
- VCF files, fastsimcoal2 input files and statistics computed over genomic windows are available from figshare (https://doi.org/10.6084/m9.figshare.c.6140793.v1).
- Bioinformatic and msprime simulation scripts are available from https://github.com/pi3rrr3/antmixture

We thank once again the Senior Editor and the Reviewers for their comments, which significantly improved our work. We also thank the editorial office for allowing us sufficient time for submitting this new version. We hope our revised manuscript meets the standards of PLOS Biology.

On behalf of the coauthors,
Pierre Nouhaud

REVIEWERS' COMMENTS:

Reviewer #1:

I enjoyed reading the manuscript, which was well written and addresses a timely question about hybridization using approaches from an evolutionary genomics perspective. The main results are that (probably) independent hybrid populations (i) show similar evolutionary sorting of ancestry, and (ii) that the tendancy for regions to be sorted is predictable based on features such as gene density, previous history of selection, and local recombination rate.

The system is compelling, the data are high quality and this quality is matched by a quality analysis. This work will clearly be the foundation for a high-quality and long-term research programme addressing the most outstanding questions in the field.

A side effect of timeliness, however, is a rapidly expanding literature among which this work will stand. Importantly, I found that this paper did not cite several recent and highly relevant articles (I am not an author on any papers referenced). In particular, recent work in swordtail fish, most importantly including Langdon et al. 2022 PLos Genetics, should probably be referenced and discussed. I would also like to see the patterns put more into the context of emerging theory, for example by Carl Veller (bioRxiv 2019 and ensuing articles).

We thank the reviewer for their kind words and for suggesting additional references. We have included them all, along with suggestions from Reviewer 2, and additional articles (Lines 38-47,52,55-61; line numbers refer to the track-changes version). We now refer in the introduction to previous work performed in sunflowers (Buerkle and Rieseberg 2008), monkeyflowers (Brandvain et al. 2014), humans (Harris and Nielsen 2016), swordtail fish (Schumer et al. 2018; Langdon et al. 2022), sparrows (Runemark et al. 2018), butterflies (Edelman et al. 2019; Chaturvedi et al. 2020) and maize (Calfee et al. 2021). Additionally, we have added mentions to Rieseberg et al. (2003, on genome stabilization), Duranton and Pool (2022, on the impact of admixture proportions) and Matute et al. (2020, on experimental admixture studies) and now discuss briefly our results in the light of theoretical work from Sachdeva & Barton (2018) and Veller et al. (2021). We have also discussed future perspectives on the identification of candidate incompatibility loci from large-scale genomic data, leveraging approaches from Blanckaert & Payseur (2022) and Li et al. (2022). The full, updated reference list can be found in the final version of our manuscript (not the track-changes version, because of PaperPile issues).

I have minor concerns about the analysis that can probably be solved with a bit more transparency. In particular I am slightly concerned that hybrid incompatibilities could be filtered out by the Hardy-Weinberg based heterozygosity filter.

Thanks to the Reviewer for pointing out their concerns. We have clarified this in the methods section L298-304 and explain the reasoning why this filtering should not remove incompatibilities. This filtering step is done after pooling all samples together (two species and three hybrid populations), which is expected to lead to population subdivision and hence a signature of Wahlund effect, with an excess of homozygotes compared to expected under neutrality in a random mating population. After pooling, sites with an excess of heterozygosity compared to neutral expectation in a panmictic population are discarded, removing incorrectly aligned paralogous regions of the genome (where two haplotypes may align, leading to an excess of heterozygous genotypes). True hybrid incompatibilities would show genotypic differences between hybridizing species, i.e., they would not be heterozygous both in hybrids and individuals from both species. Hence, sites associated with hybrid incompatibilities would not be removed by this filter, even if these regions would be heterozygous in the hybrid populations.

I'd also like to see a bit more empirical rigour about why the authors suppose the mitochondria are such good evidence for independence.

We have removed the statement that the mtDNA data “support[s] a model where each hybrid population formed independently from a small number of founders” and acknowledge an alternative, although unlikely, scenario with a single admixture event L129-131. We have expanded our rationale regarding the interpretation of the mtDNA data based on wood ant

life history and biology (namely, dependent colony founding mechanisms and poor dispersal abilities are more consistent with independent origins for populations that have differing mitotypes). In the introduction we now clarify: “Although differentiation between nests within a population is low in polygynous and supercolonial species, differentiation between populations is high, likely reflecting budding as the main dispersal mode (i.e., dispersing by foot and building a new nest in the vicinity of an already established nest, reviewed in Maeder et al. 2016). Long-distance dispersal happens via temporary social parasitism, where a single mated queen (or possibly few) enters a nest from an unrelated species executing the local queen and using the local workforce to raise her first brood (Maeder et al. 2016)” (L70-77). Our new model choice analysis also supports independent admixture events for some population pairs (results L107-118, methods L424-438; Fig. 1F) and we provide more details in our answer to the Reviewer’s comment about (the former) Line 65 below.

Finally, the authors do not really discuss or address hybrid incompatibilities outside of the first paragraph of the main text. As BDMIs are hypothesized to be a major driver of hybrid genome evolution, this seems like a rather large omission.

Indeed, the hybrid genomes are likely impacted by incompatibilities as well and there is some prior evidence for incompatibilities between the two Långholmen lineages (Kulmuni & Pamilo 2014; Kulmuni et al. 2020). We now mention it in the main text and have added these two references when introducing these lineages L92-94. We have not analyzed incompatibilities in the present work since the current sampling is not adequate to carry this task confidently. We plan to focus on detecting incompatibilities in our future work by sampling sufficient numbers of males and females within a population. However, as suggested by the Reviewer, we have acknowledged the impact of incompatibilities in hybrid genome evolution at the end of the Results & Discussion section L224-230.

Line-by-line comments:

Line 18: This is good framing for the study, but I believe this is becoming increasingly clear.

We have reworded this sentence L18.

Line 38: I would argue that an additional reason to study is that it is a way to 'kick the tires' of the genome, to see how it works. We can discover new gene functions and interactions sometimes only in hybrids.

Thanks for the suggestion, we have added this L38-39 and refer to Powell et al. 2020, where hybridization revealed an incompatibility responsible for a melanoma in swordtail fish.

Line 65: This seems like a bit of a bait-and-switch when the models are equally supported below.

We apologize to the reviewer as this was not our initial intention! We have rewritten this section, removed the statement the Reviewer was referring to, and added a new section towards the end of the introduction L70-82 on dispersal in wood ants. We have also implemented a new model choice comparison based on AIC and using a dataset with one

SNP per window (results L107-118, methods L424-438; Fig. 1F). Model choice supports independent origins (IO) as a more likely scenario for two hybrid population pairs (Bun-LånW, Bun-Pik), single origin (SO) as a more likely scenario for the two lineages in the Långholmen hybrid population (LånR-LånW, which basically share the same mitotype), and inconclusive results in the remaining comparison (Pik-LånW). We now discuss mtDNA and demographic reconstruction (including model choice) results jointly, stating L126 that taken together these results “are consistent with two alternative scenarios for the origin of these hybrid populations”, namely independent admixture events or rapid dispersal after a single admixture event. However, we conclude that the latter scenario does not fit well to model choice results and what we know of wood ant biology (as mentioned at the end of the introduction L70-77), as explained L132.

Line 66: It would seem that a reference to Molly Schumer's work (published in PNAS) where assortative mating maintains two separate sympatric hybrid populations would be appropriate here.

Thanks for pointing out our omission. There is actually indirect support for assortative mating in the Långholmen population, since previous work shows that >95% of mated queens did so with a male from their own lineage. We have edited accordingly and added two references to previous studies on wood ants L92-94.

Line 66-9: It might seem trivial, but I think referencing a paper or supplementary result showing this explicitly would be valuable, especially in light of results below that suggest this is equivocal. This is important because the extent to which the paper's findings are of interest is largely determined by the degree of independence.

We have rephrased and expanded the end of this section (L98-100, 126-135) and we also provide more context on colony establishment and dispersal in *Formica* ants at the end of the introduction L70-77.

Line 71: Seems to contrast with statement below stating that F. polyctena ancestry is "more prevalent genome-wide".

Thanks for pointing it out, we have removed the second statement L174.

Line 75: Unclear from the text whether the models also included mitochondrial haplotype.

They were not included, and we are not aware of any demographic modeling framework which would leverage both mitochondrial data and > 500,000 nuclear markers and would be suited for our data. We have clarified that SFS were built from nuclear SNPs at the beginning of this section L103.

Line 83: This paragraph is well-written and the analyses are strong.

We are delighted with the Reviewer’s comment and that this section is clearly written!

Line 100: Could migration of haploid males (who would not transmit mitochondria) perhaps be responsible? (Am not an ant expert so this might be an ignorant statement).

This is a good point, but it would require significant migration. Our demographic models did not find support for a scenario where hybrid populations would receive gene flow either from hybridizing species or other hybrid populations (S2-S11 Tables), and our new model choice results rule out migration between hybrids after admixture (L124-126, methods L424-438; Fig. 1F). Such migration would also contrast with the limited dispersal abilities of both *Formica* species. For instance, Martin-Roy, Nygård et al. (2021, https://doi.org/10.1086/715012) analyzed microsatellite data collected between 2004 and 2020 in the Långholmen population, and they failed to detect any migration over this 16-year period (despite distinct populations, such as Bunkkeri, established a few kilometers around).

Line 159: Suggest specifying whether sampled populations were taken from single-species colonies in areas of allopatry or in areas of sympatry (i.e., locations are given but this does not necessarily imply that they are allopatric).

Samples are taken from single-species colonies and Finnish samples can be considered sympatric, whereas other sampling locations in this study can be considered allopatric. We have added this information, along with sample sizes, L258-264. Thanks for the important point.

Line 193: Recent theory and empirical studies suggest that hybrid incompatibilities cause selection for positive heterozygosity (Simon et al. 2018, Evol Letters; Thompson et al. 2022, PLoS Biology). Moreover, selection against recessive incompatibilities could also leave this signature. Could the authors comment on whether this step might filter out real signal? Do these sites show biased ancestry? What is the typical heterozygosity of excluded sites? Also, could the authors provide information and a graph to see if this was affecting regions or single SNPs? I'd like to know how much data loss this specific step resulted in.

This filtering step is done by pooling samples from both species together (along with hybrids), aiming to exclude sites that show an excess of heterozygosity in the pool, compared to the expectation of neutral loci in a random mating population. As such, we are not filtering on Hardy-Weinberg equilibrium, but we only remove sites that are found in heterozygous state across most individuals of the two hybridizing species and their hybrids. Hence, this filtering step should not remove segregating or fixed dominant or recessive incompatibilities in hybrids, as those would be homozygous for different alleles in the hybridizing species. Overall, 54% of the 122,044 excluded sites are located on unanchored contigs (“assembled” into Scaffold00, which totals only ~ 22% of the genome and is transposon-rich – this scaffold was excluded for local ancestry analyses). Finally, at these excluded sites, all populations show average heterozygosities between 0.48 & 0.53 (*F. aquilonia*: 0.479, *F. polyctena*: 0.482). We have clarified this in the methods section and provided additional statistics L298-304, as suggested by the Reviewer.

Line 226: Could these SNPs be used in the 'selection' analyses?

The sweep scans performed with Raisd were carried with the full dataset (including the SNPs used for demographic inference), and we now mention this L523. Local ancestry inference was also carried out using full datasets, either phased (Loter, Twisst) or unphased (naive painting), as mentioned in the methods (L480, 485, 499).

Line 279: Are migrants haploid males, diploid females, or both?

All individuals are haploid in our simulations and we have added this information L389.

Figure 1: Panel C, K=2 is not what one expects from reading the text. The hybrid populations do appear to show minor parent ancestry and different directions for different populations.

The Reviewer is correct. The skewed admixture proportions inferred by sNMF in the K = 2 clustering analysis the Reviewer is referring to (hereafter sNMF_all) are in contradiction with additional results we obtained through other methods, and we did not include an explanation in the first version of the manuscript – apologies for this omission. We think the sNMF results did not correctly capture the genetic make-up of hybrids, since three other methods give a different and more consistent picture of the admixture proportions as explained below.
The table below shows admixture proportions for each hybrid population (Bun: Bunkkeri, Pik: Pikkala, LånW: Långholmen-W, LånR: Långholmen-R) inferred through five different methods. Note that values are averaged at the population level to ease comparisons, but individual estimates have been included in the revised S1 Table.

- sNMF_all displays the *F. aquilonia* ancestry component from the joint clustering analysis the Reviewer is referring to, as displayed in Fig. 2C with all 59 individuals (run with K = 2);
- sNMF_3pop displays the *F. aquilonia* ancestry component from the same clustering analysis (run with K = 2) using the same reduced data set, with all individuals from both hybridizing species but only one hybrid population analyzed at a time (i.e., four runs overall, done to answer this comment);
- fsc2_3pop refers to the *F. aquilonia* contribution inferred from the three-population admixture models run with fastsimcoal2, as reported in the manuscript (Fig. 1E);
- Loter is the local ancestry inference method we used in the manuscript (e.g., Fig. 2A), with reference panels comprising non-admixed individuals from both hybridizing species – values are hybrid indices ranging from 0 (non-admixed *F. aquilonia*) to 1 (non-admixed *F. polyctena*);
- Painting refers to the naive chromosome painting performed in the manuscript (S3 Fig.) using ancestry-informative markers (AIMs) displaying allele frequency differences ≥ 80% between non-admixed individuals from both hybridizing species – values are also hybrid indices ranging from 0 (non-admixed *F. aquilonia*) to 1 (non-admixed *F. polyctena*).

**Method**	**sNMF_all**	**sNMF_3pop**	**fsc2_3pop**	**Loter**	**Painting**
Data	5kbp-thinned, MAC ≥ 2		Full data	Phased data	AIMs
# SNPs	46,896		1,537,053	1,490,364	79,336
Bun	0.65	0.78	0.43	0.52	0.52
Pik	0.81	0.65	0.41	0.52	0.48
LånW	0.13	0.46	0.45	0.49	0.44

LånR	0.11	0.36	0.35	0.52	0.41

In all analyses reported above, and for all hybrid populations, admixture proportions are always more balanced with fastsimcoal2, Loter and naive chromosome painting than suggested by the sNMF_all analysis alone (apart from the Bunkkeri population in the sNMF_3pop analysis). Our PCA results (based on the same data set as our sNMF runs, Fig. 1B) also support more balanced admixture proportions in hybrids.
Taken together, these results suggest that admixture proportions inferred with the sNMF_all clustering analysis at *K* = 2 do not adequately capture the introgression patterns in the data (biases associated with clustering analyses have been documented elsewhere, see e.g. Lawson et al. 2018 https://doi.org/10.1038/s41467-018-05257-7), which could be due to the linkage structure or our sampling scheme (10 individuals per species vs. 39 hybrids in the joint sNMF_all analysis). sNMF results obtained using the same data set (i.e., same linkage structure) but with a single hybrid population at a time (sNMF_3pop) are more in line with our other methods (see table above). Moreover, the 10 sNMF iterations performed for *K* = 2 show highly variable admixture proportions in hybrids, suggesting possible convergence issues (see file enclosed, for review only). In the revised version we decided to maintain *K* = 2 results in Fig. 1C since it shows admixture in hybrids, and as mentioned in the figure legend, the cross-entropy analysis gives *K* = 6 as the optimal number of clusters. To alleviate any concern, as rightfully raised by the Reviewer, we have added individual admixture proportions and hybrid indices in the updated S1 Table (these values are averaged per population in the table above). We now refer to these results in the legend of Fig. 1 (L728-730) and in the main text L89.

Figure 2: Panel A, not immediately clear why Scaffold 13 is used. Perhaps the figure could note why it was selected. Panels C-D, I must say that I found the 'topology weighting' approach from the figure was not what I expected from the text. The approaches taken by Langdon et al. 2022 PLoS Genetics are, to my eye, more intuitive and easier to evaluate, though I must say that after spending some time with the analysis I like the weighting approach and it is sound. I think the inset Figures added to my initial confusion: for the toplogies it appears as though the 'hybrid' is a separate entity from the two parent species, when the approach is really asking which of the two parents the sequences from hybrid populations resemble. Perhaps some annotation could help here, e.g., 'hybrid population sequence similar to F. polyctena'.

We have edited the legend L747 to highlight that along the genome, hybrids are locally more related to either species depending on the color (green: hybrids locally related to *F. aquilonia*, yellow: hybrids locally related to *F. polyctena*). We have also mentioned in the legend that panels A & B were “examples of (A) local ancestry and (B) topology weighting patterns [...] inferred along one pseudo-chromosome (Scaffold 13)”. Other chromosomes show similar overall patterns and Scaffold 13 is not an exception.

Figure 3: The presentation of data in panel A is wonderful. I haven't seen it before and if the authors came up with it, I think it is quite an innovation. For Panel B it would seem to be the axes should be flipped—isn't the statement that coding SNPs predicts ancestry, not that ancestry predicts coding SNPs?

Thanks for the kind words and for suggesting flipping the axes of Panel B – this has been corrected in the updated Figure 3 and its legend (L768-769).

Figure 4: This is also a very cool result and I think quite novel. In the caption instead of 'plain' I might suggest 'solid'.

Thanks, we agree this is a quite novel result and have aimed to highlight this better in our discussion L220-230! We have edited the caption accordingly L784.

Reviewer #2:

This paper provides evidence that sorting of ancestry in hybrid ant populations is repeatable and predictable based on inferred patterns of selection and recombination rate variation. The degree of inferred repeatability (correlations >0.5 for ancestry between pairs of populations) is really remarkable. If the results hold (see my comments below) this is a really exciting study.

My main concern is that interpretation of the results depends heavily on whether (or rather to what extent) the four hybrid ant populations evolved independently (i.e., independent origins and subsequent connection versus not by gene flow). The authors are clearly aware of this as well, and several analyses are aimed at addressing the degree of independence. I appreciate this, but I am not yet fully convinced that the paper has sufficiently ruled out ancestry correlations being mostly driven by shared histories.

First, as the authors note, models of shared versus independent origins had similar likelihoods. This concern is somewhat tempered by the evidence that a modest number of generations (~20% of the time since admixture or 10 out of 50 generations) passed between admixture and population splitting in the single origin models. Still, it is not clear what proportion of the observed ancestry correlations can be explained by a single origin model with ~10 generations of shared history (I am not just interested in whether the correlations are higher than expected under such a null model, but how much higher). This is an important omission.

We thank the Reviewer for their insightful comments on our manuscript and we are glad they share our excitement about these findings. We agree this was indeed an important omission from the first version of our manuscript, as parallel patterns of enrichment we report could, in theory, have been caused by sorting in an ancestral hybrid population, before hybrid lineages diverged into the present-day hybrid populations we sampled (single origin scenario, SO). This ancestral sorting would not occur under an independent origins scenario (IO), where hybrid populations would derive from distinct admixture events. Under this IO scenario, parallel signals could arise through gene flow or through repeated selection (or a combination of both). In this revised version we disentangled between these competing hypotheses through two additional analyses.
First, to elucidate the history of these hybrid populations and ultimately reveal the contribution of shared history in the similarities among populations, we have now added an AIC-based model choice procedure, comparing the relative support towards both IO and SO

scenarios (new Fig. 1F, results and discussion Lines 107-132, methods L424-438, line numbers refer to the track-changes version). Model choice provides more support towards an IO scenario for two of the four hybrid population pairs considered: Bunkkeri - LångholmenW (median relative likelihoods: L_{IO} = 0.99, L_{SO} = 0.01) and Bunkkeri - Pikkala (L_{IO} = 0.88, L_{SO} = 0.10; Fig. 1F). The SO scenario is only supported for the two Långholmen lineages, which basically share the same mitotype and were hypothesized to originate from a single hybridization event because of their geographical location (LångholmenW - LångholmenR, L_{IO} = 0, L_{SO} = 1.00; Fig. 1F). Finally, results are inconclusive for the remaining pair (Pikkala - LångholmenW: L_{IO} = 0.43, L_{SO} = 0.37; Fig. 1F). We interpret these new results as arguing against shared history explaining the parallelism observed between two hybrid population pairs. They suggest instead that observed parallel patterns could emerge repeatedly after admixture. Model choice also consistently argues against migration between hybrid populations after admixture in all hybrid population pairs, in line with a very limited role for gene flow in the parallelism observed (independent origins with migration scenario, IOm, L_{IOm} < 0.12 in all comparisons, Fig. 1F). Taken together, these new results suggest that the observed genomic patterns emerged repeatedly and independently after admixture for at least two hybrid population pairs, while ancestral sorting could be responsible for the patterns observed for one pair (Långholmen lineages, which share similar mitotypes). We however explicitly acknowledge in the main text L133-135 that we are dealing with very recent admixture events, at the limit of coalescent-based inference with our dataset (relatively few lineages sampled, compared to a recent admixture history).
Second, to answer the Reviewer's comment on the proportion of the observed ancestry correlations which could be explained by an SO scenario (following the minor comment made by the Reviewer about L93 below), and to take into account the uncertainty between SO and IO scenarios, we now present results of neutral msprime simulations under both scenarios for each hybrid population pair comparison. Both simulation sets capture the diversity and divergence levels we observed in our dataset (see our answer to the Reviewer's next comment, S12-S13 Tables). Moreover, both SO and IO models lead to similar correlation levels in local ancestry between hybrid populations, and these simulated correlation levels match the observed ones (updated Fig. 2C-E, S4 Fig.). The results of these new analyses indicate that under realistic demographic histories, high correlation levels can be reached without any period of shared hybrid history. Note, however, that under both our neutral IO and SO simulations, the amount of sorting is always lower than observed, suggesting that selection also contributes to the similarities observed among populations (updated Fig. 2E, S4 Fig.).
We have also added information on wood ant colony establishment in the introduction (L70-82), providing more context on the patterns of mitochondrial inheritance observed in our dataset (L92-100) and their likelihoods under both IO and SO scenarios (L126-133).
We thank the Reviewer for allowing us to clarify this important point and hope that our new analyses demonstrate that (*i*) shared history can be ruled out to explain parallel sorting in two of the four population pairs considered and (*ii*) under realistic demographic histories, shared history is not required to explain high levels of parallelism.

Second, it is not clear that the best models from fastsimcoal2 are good models for the data. Specifically, the paper notes several ways in which these demographic models fail to predict patterns of ancestry (e.g., degree of sorting of ancestry). If the models do not explain the data particularly well (even if they are the best of the models considered) than it is harder to interpret deviations from predictions from the models as evidence of selection. I think two

things should be done to address this issue. First, it would be nice to see that data simulated under the best models recreate patterns of genetic variation within and among the populations in general (even if not patterns of ancestry).

The Reviewer is absolutely right and we warmly thank them for their comment. It allowed us to identify an error in our msprime simulations, which results were initially displayed in our previous Figure 2E. The N_e estimates used in our msprime simulations were not rescaled properly (rescaling was required to account for ploidy level differences between fastsimcoal2 and msprime) and initial msprime simulations were run with larger N_e estimates than the demographic modeling assumed. This error has been fixed and the conclusions of our study remain unaltered. Now, for each pairwise hybrid population comparison we present results of simulations under both independent and single origin models (S3-4 Fig.). We also now provide diversity (π, S12 Table) and divergence (pairwise F_{ST}, S13 Table) metrics for each simulated population, as suggested by the reviewer. For both single and independent origins scenarios, these simulated estimates are close to the observed values. Finally, we show that our simulations adequately capture the structure of the dataset by displaying the output of PCAs for one run per simulated model (S5 Fig.).

Second, and especially if the models do not appear to predict genetic variation well, I think demographic models (especially models for single versus multiple origins) should be fit for the ancestry data directly (perhaps from the simple, naive chromosome painting, but any would be fine). It would be very interesting to see whether such models would suggest a single origin with more shared history.

While this would be an amazing tool for the study of hybridization, we are not aware of any framework which would allow fitting such demographic models directly on the output of ancestry inference methods. This would require taking into account the ascertainment schemes associated with most ancestry inference pipelines (e.g., in this work the naive chromosome painting data only considers sites displaying an allele frequency difference of at least 80% between the two species, thus using only 5% of the data available).

My other more minor comments (with approximate line numbers) follow:

L35-45. I appreciate the brevity of the introductory paragraph but think that it consequently has fallen a bit short on putting the work in context. Most notably, this is not the first study to look at patterns of genome sorting (genome stabilization) in hybrid lineages. Perhaps the best example concerns hybrid sunflowers (see, e.g., https://doi.org/10.1111/j.1558-5646.2007.00267.x; DOI: 10.1126/science.1086949), but other examples that consider repeated instances of hybrid lineage formation (Italian sparrows, Lycaeides butterflies) or that explicitly consider selection and recombination (swordtail fish) exist. This doesn't all need to be cited, but some reference somewhere to the relevant literature would be good. Likewise, the references in this section are all from the past few years but thinking on this topic goes back decades. Lastly, the term genome stabilization has been used in many papers to refer to what is here called genome sorting. I don't have a strong preference for one term over the other, but the connection should be made parenthetically.

Thanks for suggesting additional references, we have now included all of them in the main text. We have edited the introduction following your remarks L38-47 (along with suggestions from Reviewer 1) and now refer to previous findings in sunflowers (Buerkle and Rieseberg 2008), monkeyflowers (Brandvain et al. 2014), humans (Harris and Nielsen 2016), swordtail fish (Schumer et al. 2018; Langdon et al. 2022), sparrows (Runemark et al. 2018), butterflies (Edelman et al. 2019; Chaturvedi et al. 2020) and maize (Calfee et al. 2021). Regarding repeated instances of admixture, we now explicitly refer to Runemark et al. (2018), Chaturvedi et al. (2020) and Langdon et al. (2022) L58, along with discussing their results L201-219. We also linked to genome stabilization and referred to Rieseberg et al. (2003) when introducing sorting L55 – thanks for pointing out this connection. The full, updated reference list can be found in the final version of our manuscript (not the track-changes version, because of PaperPile issues)

L55-58. I think it is a bit strong to refer to this as "an ideal test case" when the evidence is only that the populations "may have independent origins" (an ideal case would be if there was very strong evidence or near certainty of independent origins).

This is a fair point – we have removed "ideal" from this sentence L81.

L89. I recommend reporting the range of correlations here. They are high and worth calling out in the main text (just stating that the correlation is significantly different than 0 is much less interesting).

Indeed, correlations range from 0.51 to 0.62 and we have added this in the main text L144.

L93. Consider simulating not just from the best model, but from several of the best competing models.

Thanks for the suggestion. For each comparison we now present results of simulations performed under both independent (IO) and single origin (SO) models in the new S3 Figure. We show that as expected, simulated correlations levels under IO are lower than under SO (this difference in correlation levels is stronger in the comparison between Långholmen lineages). Both simulated scenarios show correlation levels slightly lower than or comparable to the ones observed in the dataset (apart from the comparison between Långholmen lineages, where observed correlation levels match levels obtained under single origin simulations). However, neither IO or SO models capture the extent of sorting we observe (S3 Fig, updated Fig. 2C-E).

L112-125. These patterns of enrichment are quite cool. They definitely increase my confidence that selection has contributed to the patterns of observed ancestry, though they still don't rule out a major role for shared history for the similarities among populations.

As it was already pointed out by the Reviewer in their first comment, we agree that in the initial version of our manuscript, the parallel patterns of enrichment we report could in theory have been caused by sorting in an ancestral hybrid population. We hope that our new AIC-based model choice analysis (new Fig. 1F, results and discussion Lines 107-132, methods L424-438), additional simulations (S3 Fig.), and biological context on wood ant

colony establishment (L70-82) convinced the Reviewer that shared history can be ruled out as a driver of the parallelism observed for at least two population pairs.

L179. State what species the reference genome is for here.

We used the *F. aquilonia* × *polyctena* reference genome from Nouhaud et al. (2022) as a reference and have added this information L280. This genome was assembled from a single male sampled in the Långholmen population. Both hybridizing species show comparable mapping rates against this assembly (see Fig. 1 from Nouhaud et al., 2022; https://doi.org/10.1093/jhered/esac019).

L184. Please define "normalized" in this context.

We have added a short explanation L288. Freebayes is a haplotype-aware variant caller which outputs multi-nucleotide variants (MNVs). The normalization step consists in left-aligning these MNVs parsimoniously, so that they can be decomposed afterwards in as few SNPs as possible.

Reviewer #3:

This manuscript assesses the repeatability of genetic outcomes of hybridization in four Finnish populations of ants (in three localities), each with mixed ancestry from the widespread species Formica aquilonia and Formica polyctena. The authors find that each of the hybrid populations has fixed F. aquilonia ancestry in some parts of the genome and F. polyctena ancestry in others, and that the same genomic regions tend to be fixed for the same parental species ancestry across multiple hybrid populations . Additionally, genomic regions with low recombination rate and high gene density tend to fix alleles from the parent species with the larger inferred population size. Taken together, this suggests that natural selection shapes the similar outcomes of hybridization in multiple hybrid populations. The work addresses a consequential question in the field of speciation genetics , and the writing and figures are clear and easy to follow.

There are two areas where I am not fully convinced: First, the independence of the three localities with hybrid populations seems a bit overstated as currently written. As the authors note, the likelihoods of models assuming a single origin of hybrids vs. independent origin of each hybrid population are similar. The main text doesn't point out that the likelihoods are higher for single origin than independent origin in all four pairwise comparisons of hybrid populations, suggesting that the histories of these Finnish hybrid populations are not completely independent of each other. The argument in the text that mitochondrial haplotype networks support three independent origins of hybridization doesn't appear very strong; there is substantial mitochondrial polymorphism in both parent species, and some of this variation could have been present in a single ancestral hybrid population before fixing different alleles in different descendant hybrid populations.

We thank the reviewer for their kind comments and pinpointing where more justification was needed. In the new version, we aimed at clarifying the main text by adding information on

wood ant life history and biology as well as implementing a new model choice comparison based on AIC and using a dataset with 1 SNP per window. Briefly, regarding likelihoods of independent origin (IO) and single origin (SO) models, we have added this model choice procedure in the revised version of our manuscript (new Fig. 1F, results and discussion Lines 107-132, methods L424-438, line numbers refer to the track-changes version). Model choice supports independent origins (IO) as a more likely scenario for two hybrid population pairs (Bun-LånW, Bun-Pik), single origin (SO) as a more likely scenario for Långholmen lineages (LånR-LånW, which both share similar mitotypes), and inconclusive results in the remaining comparison (Pik-LånW). While we are dealing with very recent admixture events, at the limit of coalescent-based inference with our dataset (relatively few lineages sampled, compared to a recent admixture history), our model choice procedure based on a set of potentially unlinked loci also consistently argues against independent origins with migration in all comparisons (Fig. 1F). To take into account the uncertainty between SO and IO scenarios, we have now simulated the correlation and sorting levels expected with both scenarios under neutrality (S3-4 Fig.). In all cases, our simulations match the empirical dataset (diversity estimates in S12 Table, divergence estimates in S13 Table, population structure in S6 Fig.) and show that while high correlation levels between admixture levels could be reached under neutrality alone, <u>observed levels of sorting cannot</u>, and this no matter the level of independence between hybrid populations (Fig. 2C-E).
Regarding patterns of mtDNA inheritance, we have now edited the introduction to provide more biological context on colony establishment in wood ants (L70-82) and discuss together results from mitotype network and admixture history reconstruction L126-135. To highlight the divergence observed in the mtDNA dataset, we have also indicated on the mitotype network edges the number of mutational steps which are greater than or equal to five (Fig. 1D). Excluding the two admixed *F. polyctena* individuals (identified with arrows on Fig. 1D), 108 out of 199 mitochondrial SNPs (54%) discriminate between both species. While we agree with the Reviewer that “there is substantial mitochondrial polymorphism in both parent species”, sampling of these individuals was carried across Europe (Fig. 1A), and the canonical wood ant supercolony usually only carries a single or a few mitotypes (Beresford et al. 2017, https://doi.org/10.1111/mec.14183). It is thus highly unlikely such mtDNA polymorphism segregated in an ancestral hybrid population.
We hope these edits will provide the Reviewer with a more detailed picture of the level of independence between hybrid wood ant populations in our study.

A more substantial logical inconsistency is that the study relies heavily on analyses that assume neutrality in order to argue for pervasive effects of selection across the genome. To what extent and in what direction would the inferences of demographic history and recombination be biased by the selection required to produce the observed levels of sorting of parental variation in the hybrid populations, and the number and extent of inferred selective sweeps in the parent species? The demographic results, in particular, would be more convincing if similar results are obtained when restricting the analysis to genomic regions that are not sorted in hybrids, and/or have low [mu] scores in the scan for signatures of selection. In fact, convergent selection in hybrid populations with truly independent origins might even produce a false signal of shared ancestry or gene flow.

This is a very good point, and we followed the Reviewer’s suggestion by re-running all our four-population demographic models after excluding SNPs located in sorted regions (as

defined in our manuscript: 20 kb windows displaying ≥90% of a single ancestry component in all 4 populations, ca. 1.1 million SNPs remaining).
Unfortunately, these additional analyses did not help us to disentangle between SO and IO scenarios. For each pair of populations, IO and SO models had comparable likelihoods with this reduced dataset. However, many parameter estimates were not consistent across population pairs (e.g., admixture proportions, N_e estimates), suggesting potential convergence issues in the fastsimcoal2 algorithm with the reduced dataset. Discarding sorted regions resulted in on average a 37% reduction in the total number of sites remaining to build SFSs (number of sites with vs. without sorted regions; Bun-Pik: 328,913 vs. 206,214, Bun-LånW: 282,215 vs. 176,497, Pik-LånW: 218,545 vs. 137,311, LånW-LånR: 150,207 vs. 93,846). For this reason, and since the overall conclusions were not different from the full dataset, we decided against including these new analyses in the revised manuscript.

Minor details:

L386: change "was ran" to "was run"

Corrected, thanks (L514).

Tables S3 and S4 have Excel formula errors, such that Δlikelihood and LogLikelihood are identical for four of the models in each table.

Thanks for spotting this error – values have been corrected.

Reviewer #4:

This paper presents fascinating findings. Namely, that three separate hybridization events have occurred between two closely related species of ants, and that each hybrid population tends to share similar ancestry components along the genome. They suggest this pattern of correlated ancestry component sorting cannot be explained by chance and that shared selection pressure for specific ancestry components is the best explanation. I found the paper to be well-written and their approach and methods to be sensible and clearly justified. In particular, the authors take considerable efforts to model and account for demographic history and recombination rate variation, both of which effect signatures of selection and hybridization.

Despite very much liking the paper overall, I have some criticisms related to how the authors presented and used simulations to support their claims.
In the results section on line 90, the authors state: "To test whether such predictability would be expected under neutrality, we used MSPRIME (15) to simulate neutral admixture scenarios following the best history and demographic parameters inferred for each population pair"

Related to this in the methods section on line 380 they state: "To do so, we used the parameters inferred by the best model identified for each population pair with

FASTSIMCOAL2 and ran simulations in MSPRIME (v1.0.2, 15), modeling 100 non-recombining 10 kbp blocks with a recombination rate of 10-6 within blocks."

This is more or less all the details readers are given in the text about the msprime simulations, and it is difficult for me to tell what exactly was done. If I understand correctly, the authors fit models with FASTSIMCOAL2 that included all their populations. Did the msprime simulations exactly match the models inferred in FASTSIMCOAL2? Were all populations and their demographic changes and sizes included in these simulations, or were simulations done separately for different pairs? It seems quite important to me that the authors did the former in order to make apples to apples comparisons to their empirical results, but I cannot quite tell from these sentences if that is what they did, or if simpler simulations with two populations at a time were considered. Please better clarify what was done and justify these decisions in the text.

We thank the reviewer for their overall positive assessment of our manuscript and for rightfully highlighting the lack of details about the msprime simulations, which we addressed in this revised version.
We ran msprime simulations independently for each hybrid population pair, using model parameters inferred for the pair by fastsimcoal2 in terms of divergence and admixture times, N_e estimates, population size changes, migration rates and admixture proportions.
To answer the reviewer comment we attempted to consider a model with five populations, using a 5D joint-SFS dataset including three hybrid populations (excluding the LånR lineage, since model choice and identical mitotype clearly support the SO scenario for the LånW-LånR pair, Fig. 1F) and the two hybridizing species (214,855 polymorphic sites). We considered a unique model where parameter estimates could point towards single or independent origins. Briefly, we assumed that after one initial admixture event (forward in time), each hybrid population could receive an independent contribution from each hybridizing species and/or from other pre-existing (ancestral) hybrid populations. If contributions from the pre-existing hybrid populations were estimated to be large (e.g., above 0.90), and contributions from hybridizing species were reduced (e.g., less than 0.10), this would point towards a single origin scenario (SO). In contrast, if contributions from hybridizing species were high (summing to more than 0.90) and contributions from pre-existing hybrid populations were low (e.g. less than 0.10), this would support an independent origins scenario (IO). Unfortunately, this approach was inconclusive (no clear support for either IO or SO), likely due to convergence issues in fastsimcoal2 caused by the attempt to estimate a large number of parameters (14) coupled with the high dimensionality of the joint 5D-SFS with many zero entries (3067 of the 6561 total entries of the SFS were empty, 47%). Thus, we decided against including these results in the revised version of our manuscript.
We however think that our pairwise approach is sound given the structure of our dataset and since many of the analyses to investigate sorting were done in a pairwise way, especially the analyses based on simulations with msprime. The msprime simulations for a given population pair were obtained using the exact history inferred from the same pair. We have now clarified the main text in the methods section Lines 506-519 (line numbers refer to the track-changes version) and made available the msprime scripts through github (https://github.com/pi3rrr3/antmixture).

Second and more importantly, given the similar fit of the shared and independent origins models (as explained on line 75), the authors need to assess the correlated sorting of ancestry from simulations for both model types. Does a model assuming shared origins have correlations close to the observed data, or does selection still need to be invoked to explain the observed patterns, even if the shared origins model is actually the correct one?

This is a very good point, and following the Reviewer's suggestion we now present results of simulations performed under both IO and SO models for each comparison (Fig. 2C-E, S4-5 Fig.). We show that simulated correlations levels under SO are slightly higher than under IO, as expected (the difference between the IO and SO models in the degree of correlation is stronger in the comparison between Långholmen lineages). Both simulated scenarios show correlation levels slightly lower than or comparable to the ones observed in the dataset (apart from the comparison between Långholmen lineages, where observed correlation levels match levels obtained under SO simulations). Interestingly, while correlation levels may be comparable, the amount of sorting was still lower in simulations than observed, suggesting neither demographic model could take such sorting into account under neutrality.

Related to my comments above, using msprime simulations that include all populations simultaneously (rather than pairs) seems important to adequately represent a shared hybrid origins model. Demonstrating that the correlation in ancestry along the genome is due to selection sorting the ancestry components, even if the inferred shared hybridization event model is actually correct, would greatly aid the main (and most exciting) claims of the paper.

As stated in our answer to the Reviewer's first comment above, our pairwise approach makes sense to model shared ancestry, since msprime simulations for a given hybrid population pair were obtained using the exact history inferred from the same hybrid population pair. This being said, reconstructing the (certainly very recent) admixture history of all populations jointly with fastsimcoal2 would require sampling many more lineages. We however agree with the Reviewer that confirming the role of selection in sorting ancestry components is an exciting endeavor, which may require more knowledge about the targets of selection (e.g., number, distribution, selection coefficients) and extensive forward simulations.
